# Liquid Chromatographic Enantioseparation of Newly Synthesized Fluorinated Tryptophan Analogs Applying Macrocyclic Glycopeptides-Based Chiral Stationary Phases Utilizing Core-Shell Particles

**DOI:** 10.3390/ijms25094719

**Published:** 2024-04-26

**Authors:** Dániel Tanács, Róbert Berkecz, Zsolt Bozsó, Gábor K. Tóth, Daniel W. Armstrong, Antal Péter, István Ilisz

**Affiliations:** 1 Institute of Pharmaceutical Analysis, University of Szeged, H-6720 Szeged, Hungary; tanacs.daniel@szte.hu (D.T.); berkecz.robert@szte.hu (R.B.); apeter@chem.u-szeged.hu (A.P.); 2 Department of Medical Chemistry, University of Szeged, H-6720 Szeged, Hungary; bozso.zsolt@med.u-szeged.hu (Z.B.); toth.gabor@med.u-szeged.hu (G.K.T.); 3 Department of Chemistry and Biochemistry, University of Texas at Arlington, Arlington, TX 76019-0065, USA; sec4dwa@uta.edu

**Keywords:** fluorinated tryptophans analogs, enantioselective separation, core-shell particles, macrocyclic glycopeptide-based chiral stationary phases

## Abstract

Due to the favorable features obtained through the incorporation of fluorine atom(s), fluorinated drugs are a group with emerging pharmaceutical importance. As their commercial availability is still very limited, to expand the range of possible candidates, new fluorinated tryptophan analogs were synthesized. Control of enantiopurity during the synthesis procedure requires that highly efficient enantioseparation methods be available. In this work, the enantioseparation of seven fluorinated tryptophans and tryptophan was studied and compared systematically to (i) develop analytical methods for enantioselective separations and (ii) explore the chromatographic features of the fluorotrytophans. For enantioresolution, macrocyclic glycopeptide-based selectors linked to core-shell particles were utilized, applying liquid chromatography-based methods. Application of the polar-ionic mode resulted in asymmetric and broadened peaks, while reversed-phase conditions, together with mobile-phase additives, resulted in baseline separation for all studied fluorinated tryptophans. The marked differences observed between the methanol and acetonitrile-containing eluent systems can be explained by the different solvation abilities of the bulk solvents of the applied mobile phases. Among the studied chiral selectors, teicoplanin and teicoplanin aglycone were found to work effectively. Under optimized conditions, baseline separations were achieved within 6 min. Ionic interactions were semi-quantitatively characterized and found to not influence enantiorecognition. Interestingly, fluorination of the analytes does not lead to marked changes in the chromatographic characteristics of the methanol-containing eluents, while larger differences were noticed when the polar but aprotic acetonitrile was applied. Experiments conducted on the influence of the separation temperature indicated that the separations are enthalpically driven, with only one exception. Enantiomeric elution order was found to be constant on both teicoplanin and teicoplanin aglycone-based chiral stationary phases (*L* < *D*) under all applied chromatographic conditions.

## 1. Introduction

Drug candidates having one or more fluorine atoms have become common. The special nature of fluorine alters a variety of the properties of certain drug molecules, conferring features including enhanced stability, more selective reactivity, and increased binding interactions. This feature of the fluorine atom offers the possibility of its incorporation into proteinogenic amino acids or biologically active peptides to obtain more favorable drug properties [1,2,3]. The improved pharmacokinetic and physicochemical properties (e.g., higher permeability, decreased clearance, increased potency, etc.) obtained from fluorine substitution give these drug candidates very high pharmaceutical importance [4,5]. Fluorine-containing pharmaceuticals have accounted for ca. 20% of commercialized medications in recent years [5]. All the potential improvements in biological activity achieved by the incorporation of fluorine have inspired organic chemists to develop fluorinated molecules with various structures. Despite advances in the development and use of fluorinated compounds, relatively few fluorinated amino acid derivatives are commercially available. One very promising fluoro-amino acid family would be the fluorinated tryptophan derivatives.

Before their incorporation into peptides, amino acids are protected most frequently by tert-butyloxycarbonyl (Boc) or 9-fluorenylmethoxycarbonyl (Fmoc) groups. This procedure and peptide synthesis are often accompanied by racemization [6,7]. Moreover, after peptide synthesis, the presence of amino acids in the prepared peptides is generally determined after acidic hydrolysis, which also may lead to racemization. As a result of these possible transformations, it is necessary to have access to enantiomerically pure substances and effective analytical methods for the enantioseparation and identification of fluorotryptophan analogs. Of the available analytical methods, high-performance liquid chromatography is most widely used for the separation and quantitation of stereoisomers. The various possibilities for stereoselective analyses have been reviewed in several papers and book chapters [8,9,10,11,12,13,14,15,16].

Despite its potential importance, relatively little information is available on the separation of liquid chromatography-based enantioselective resolution of fluorinated amino acids. For the separation of fluorine-substituted phenylalanine analogs, ligand-exchange micellar capillary chromatography was applied [17], while separation of stereoisomers of nonproteogenic polyfluoroamino acids and peptides was carried out on a Chiralcel OD-H column [18]. Similarly, polysaccharide-based chiral stationary phases (CSPs) were applied for the enantioseparation of five -NHBoc and -CO_2_Et-protected fluorinated cyclic *β^3^*-amino acid analogs [19]. Very recently, macrocyclic glycopeptide and cyclofructan-6 [20], and *Cinchona* alkaloid-based zwitterionic CSPs [21] have been successfully utilized for the enantioseparation of fluorinated *β*-phenylalanine derivatives.

Core-shell particles (superficially porous particles, SPPs) and sub-2 µm fully porous particles (FPPs) are expected to provide effective separation of a variety of chiral molecules in ultra-high-performance liquid chromatography (UHPLC). Due to the development of column technology, the most frequently applied chiral selectors were bonded to SPPs or FPPs, resulting in very effective CSPs. Macrocyclic glycopeptides (teicoplanin, teicoplanin aglycone, and vancomycin) were immobilized by Armstrong et al. [22] and Gasparrini et al. [23], while functionalized cyclofructans were linked to SPP and FPP support by Armstrong et al. [24]. SPP and FPP support polysaccharide derivatives were first immobilized by Chankvetadze et al. [25], while *Cinchona* alkaloid-based mono- and zwitterionic CSPs were developed by Armstrong et al. [26] and Lämmerhofer et al. [27], respectively. In addition to the above citations, some recent review papers have summarized these new results [28,29].

To control enantiopurity during the synthesis of fluorine-containing amino acid analogs, highly efficient enantioseparation methods are needed. This work aims to explore the possibilities for the direct enantioseparation of newly synthesized fluorinated tryptophan analogs, utilizing macrocyclic glycopeptide-based selectors covalently bonded to core-shell particles. Liquid chromatographic measurements were performed in the reversed-phase mode (RPM) and polar-ionic mode (PIM), utilizing CSPs based on vancomycin, teicoplanin, and teicoplanin aglycone. To obtain information on the enantiorecognition processes, effects of chromatographic conditions, such as mobile-phase additives, eluent composition, nature of selector, temperature, and column dimensions were studied systematically. Based on the structural properties of the studied analytes and selectors and the knowledge of the absolute configuration of all the studied enantiomers, general trends of the chiral-recognition process could be identified and explained. We strongly believe that the results obtained in this study can serve as a basis for further developments aiming at the enantioseparation of fluorinated amino acid analogs.

**Figure 1:**
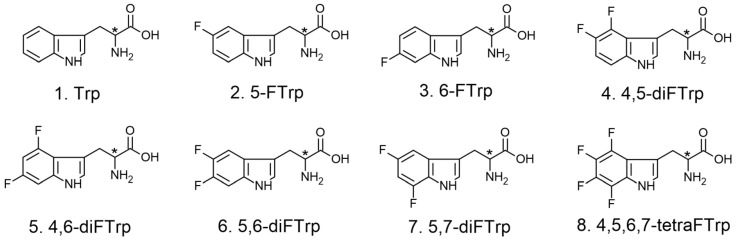
Structures of analytes. **1.** tryptophan (Trp); **2.** 5-fluorotryptophan, (5-FTrp); **3.** 6-fluorotryptophan (6-FTrp); **4.** 4,5-di-fluorotryptophan (4,5-diFTrp); **5.** 4,6-di-fluorotryptophan (4,6-diFTrp); **6.** 5,6-di-fluorotryptophan (5,6-diFTrp); **7.** 5,7-di-fluorotryptophan (5,7-diFTrp); **8.** 4,5,6,7-tetra-fluorotryptophan (4,5,6,7-tetraFTrp).

**Figure 2:**
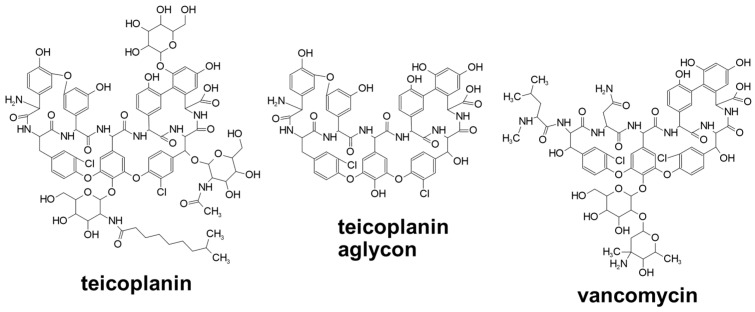
Structures of selectors.

The structures of the studied analytes are shown in Figure 1, while the structures of the chiral selectors are illustrated in Figure 2.

## 2. Results and Discussion

### 2.1. Influence of Mobile-Phase Additives on Chromatographic Performance

Macrocyclic glycopeptide-based CSPs applying either RPM with MeOH or MeCN and aqueous acid or salt additives or PIM with MeOH/MeCN as a bulk solvent, often in the presence of triethylammonium acetate (TEAA), offer efficient enantioseparation of various types of analytes [20,30,31,32,33,34,35]. First, to explore the possible effects of mobile-phase additives, NH_4_HCOO, NH_4_OAc, TEAA, FA, or AcOH was added to the eluent and the chromatographic performance was recorded for one monofluoro- (5-FTrp; **2**) and one difluoro- (5,6-diFTrp; **6**) tryptophan. In these experiments, the vancomycin-based V-3.0 CSP exhibited only moderate enantiorecognition ability, while the teicoplanin-based T-3.0 and teicoplanin aglycone-based Tag-3.0 CSPs provided better results in both RPM and PIM. To illustrate the effects of the additives, chromatograms recorded for analytes **2** and **6** in RPM are shown in Figure S1. Among organic salts (NH_4_HCOO, NH_4_OAc, TEAA), only TEAA resulted in acceptable peak shapes. In contrast, when FA or AcOH was used as a mobile-phase additive, better peak shapes and higher resolution could be obtained. In general, the best peak shapes, selectivities, and resolutions were observed with AcOH; therefore, in further experiments, AcOH was the favored mobile-phase additive. Additionally, it should be noted that the use of AcOH as a mobile-phase additive may also provide higher sensitivity when mass spectrometric detection is applied [20]. It is worth mentioning that (a) chiral recognition in RPM without any additives was possible but resulted in tailing peaks and shifting retention times, probably due to the lack of constant ionic strength; and (b) the very commonly applied PIM employing MeOH/MeCN bulk solvent in the presence or absence of any additive yielded asymmetric and very broad peaks for the fluorotryptophans; therefore, all further experiments were carried out in RPM.

### 2.2. Influence of Eluent Composition on the Chromatographic Performance

**Figure 3:**
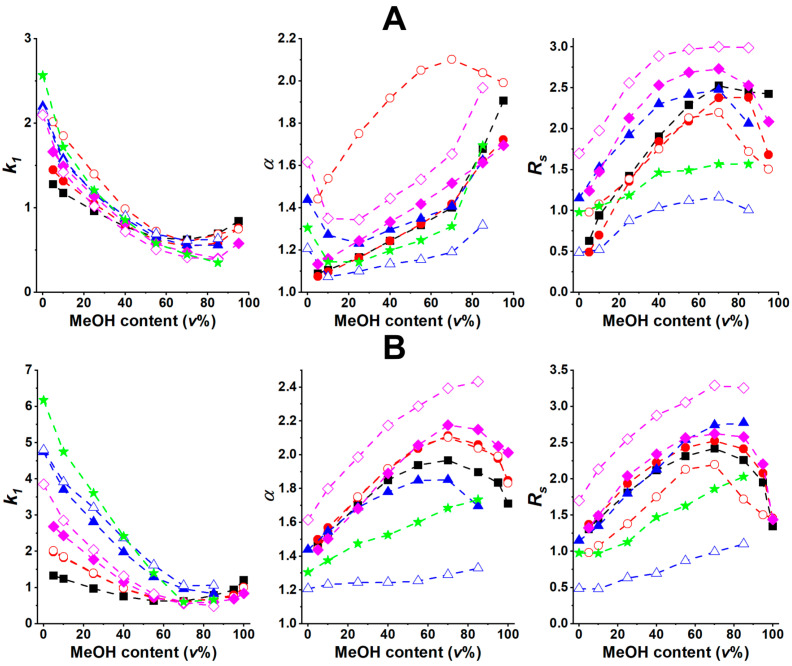
Effect of mobile-phase composition on chromatographic parameters. Chromatographic conditions: column, (**A**), TeicoShell, (**B**), TagShell; mobile phase, H_2_O/MeOH = 100/0–0/100 (*v/v*) containing 0.1 *v*% AcOH; detection, 215 nm; flow rate, 0.3 mL min^–1^; temperature, 20 °C; symbols for analytes, Trp, ■; 5-FTrp, ●; 6-FTrp, 〇; 4,5-diFTrp, ▲; 4,6-diFTrp, △; 5,6-diFTrp, ◆; 5,7-diFTrp, ◇; 4,5,6,7-tetraFTrp, ★.

Variation in mobile-phase composition is the simplest way to explore the retention properties of the newly synthesized fluorotryptophan analogs. First, the H_2_O/MeOH ratio was varied from 100/0 to 0/100 (*v/v*) (where all eluents contained 0.1 *v*% AcOH) and the chromatographic characteristics of all the studied analytes were determined. As depicted in Figure 3, effective separations could be achieved with the T-3.0 and Tag-3.0 columns.

The retention behaviors of the fluorotryptophans were similar on both columns; a curve with a slight minimum could be registered, and *k*_1_ increased with increasing H_2_O content for all the analytes. Interestingly, at very high MeOH levels, a slight increase in retention could be detected, below ca. 20 *v*% of H_2_O. The effect of the MeOH/H_2_O ratio on the chromatographic separations can be interpreted to be a result of changes in mobile-phase polarity. An increase in *k*_1_ with increasing H_2_O content relates to increasing hydrophobic interactions between the selector and amino acids. On the other hand, the solvation of the polar amino acids and their solubility decreases at high MeOH content, resulting in a slight increase in *k*_1_.

**Figure 4:**
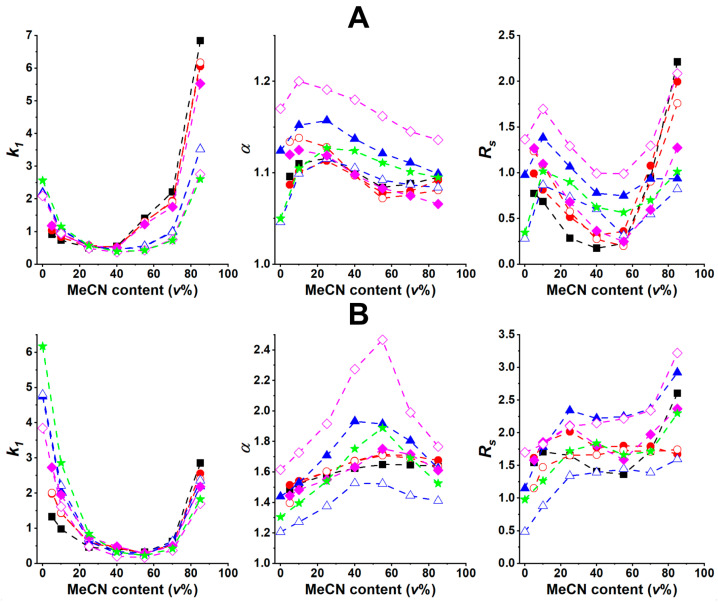
Effect of mobile-phase composition on chromatographic parameters. Chromatographic conditions: column, (**A**), TeicoShell, (**B**), TagShell; mobile phase, H_2_O/MeCN = 100/0–15/85 (*v/v*) containing 0.1 *v*% AcOH; detection, 215 nm; flow rate, 0.3 mL min^−1^; temperature, 20 °C; symbols for analytes, Trp, ■; 5-FTrp, ●; 6-FTrp, 〇; 4,5-diFTrp, ▲; 4,6-diFTrp, △; 5,6-diFTrp, ◆; 5,7-diFTrp, ◇; 4,5,6,7-tetraFTrp, ★.

In another set of experiments, RPM systems based on the utilization of MeCN were also studied. In H_2_O/MeCN = 100/0–15/85 (*v/v*) containing 0.1 *v*% AcOH mobile phases retention curves with a more pronounced minimum (observed between 40–60 *v*% MeCN content) were detected on both columns (Figure 4). Compared to the above-discussed H_2_O/MeOH eluents, a much greater increase in the *k*_1_ values was registered above 70 *v*% MeCN content. The difference observed between the two mobile-phase systems can mainly be attributed to the different solvation properties of MeCN. The solubility of ionized analytes is less favored in polar but aprotic MeCN than in the protic MeOH, resulting in a more significant increase in retention at high (>70 *v*%) MeCN contents.

From a comparison of the chromatographic parameters illustrated in Figure 3 and Figure 4, interesting findings can be identified. In the MeOH-containing eluent systems, enantioselectivity exhibited a minimum value on the T-3.0 column at ca. 10 *v*% MeOH content, then it increased with increasing MeOH content for most of the analytes (the only exception being analyte **3**, which reached an enantioselectivity maximum at ca. 70 *v*% MeOH content). On the Tag-3.0 column, a marked increase in *α* can be observed with increasing MeOH content, with α reaching a maximum value at 70–80 *v*% MeOH content (Figure 3B). In the MeCN-containing mobile phases, *α* exhibited a maximum value on both columns at between 10–30 *v*% (Figure 4A) and 50–60 *v*% (Figure 4B) MeCN content on T-3.0 and Tag-3.0, respectively.

Regarding the effect of eluent composition on resolution, it can be concluded that in the MeOH-containing mobile phases, *R_S_* on both columns reaches a maximum at 70–80 *v*% MeOH, which aligns with the eluent composition that results in maximum *α* and minimum *k*_1_, making it very favorable from the chromatographic point of view (Figure 3). In the MeCN-containing mobile phases a different behavior was observed. After some initial increase, a decrease, then another increase in *R_S_* can be observed on both columns (Figure 4), suggesting that column kinetics has a more complex influence on chromatographic performance compared to the MeOH-containing eluents.

TEAA is a commonly used mobile-phase additive in chiral separations achieved with macrocyclic glycopeptide-based CSPs in PIM [20,31,32]. To explore the possible effects of applying 0.1 *v*% TEAA (instead of 0.1 *v*% AcOH), both H_2_O/MeOH and H_2_O/MeCN mobile phases were studied with T-3.0 and Tag-3.0 columns. Results obtained with selected analytes (**1**, **2**, **3**, and **6**) indicate that the general trends observed in the changes in *k*_1_, *α*, and *R_S_* values as a function of eluent composition (depicted in Figures S2 and S3) are highly similar to those observed in the presence of AcOH (Figure 3 and Figure 4), i.e., *k*_1_ exhibited a minimum, while *α* and *R_S_* exhibited a maximum with increasing MeOH or MeCN content. It is worth mentioning that considerably lower retention times were obtained with TEAA, while selectivity and resolution in most cases were slightly lower or comparable. Considering the negative impact of TEAA on mass-detection sensitivity, the use of AcOH as a mobile-phase additive is highly recommended.

### 2.3. Evaluation of the Role of the Ionic Interactions

As macrocyclic antibiotic-based selectors contain ionizable functional groups, ionic interactions may play an important role in enantiorecognition processes [20,36]. Selectors based on teicoplanin or teicoplanin aglycone contain carboxyl and primary amino groups available for ionic interactions with the amino acids present in their zwitterionic forms in the aqueous eluents. To gain deeper insight into the details of the retention mechanism and to evaluate the role of the ionic interactions, we applied the so-called stoichiometric displacement model [37]. The model establishes a linear correlation between the logarithm of the retention factor and the logarithm of the concentration of the counterion, as described by Equation (1), below:

(1) $$log\;k=\log\;K_{Z}-Z\;log\;c_{counterion}$$

where *K_z_* describes the ion-exchange equilibrium and *Z* is the effective charge. To explore the possible effects of the ionic interactions in the case of T-3.0 and Tag-3.0 CSPs, the concentration of AcOH (counterion) was varied in the range of 2.5–80 mM (i.e., 2.5, 5.0, 10.0, 20.0, 40 and 80.0 mM) in the optimized H_2_O/MeOH = 30/70 (*v/v*) mobile phase. (The *pK* values of the studied analytes were calculated with Marvin Sketch v. 17.29 software, ChemAxon, Budapest, and are illustrated in Table S1.) As shown in Figure S4, with increased counterion concentration, reduced retention is obtained for all the analytes. The square of the correlation coefficient of the linear fittings is greater than 0.96 in all cases, while the slopes vary between −0.14 and −0.18 and between −0.17 and −0.21 in the case of the T-3.0 and Tag-3.0 columns, respectively. Based on the obtained results, two important conclusions can be drawn: (i) the small slope values indicate that the ion-exchange process does not play a determining role in the retention of the fluorinated amino acids, and (ii) the similarity of the slopes calculated for the single enantiomers indicates that the ion-exchange process affects retention but has no vital role in chiral recognition. Similar experiments carried out with TEAA as a counterion led to a similar conclusion, i.e., ionic interactions have no marked influence on the recognition mechanism.

### 2.4. Influence of the Structure of the Analyte and Selector on Retention and Selectivity

The complex structure of the macrocyclic antibiotic-based selectors and their multiple functional groups enable them to form a wide range of interactions (e.g., electrostatic, hydrophobic, π-π, H-bridge, steric hindrance, etc.) [36]. Their ability for enantiomer recognition is also influenced by the ability of the selectand to fit the hydrophobic basket of the chiral selector [36]. To explore the influence of the structures of the analyte and selector on retention and selectivity, *k*_1_, *α*, and *R_S_* values were evaluated on T-3.0 and Tag-3.0 columns with two mobile phases of similar eluent strength, i.e., H_2_O/MeOH = 30/70 (*v/v*) (Table 1) and H_2_O/MeCN = 45/55 (*v/v*) (Table 2); both contained 0.1 *v*% AcOH.

Based on the data presented in Table 1 for the MeOH-containing mobile phases, the following conclusions can be drawn: (i) monofluoro substitution has only a slight effect on *k*_1_, *α*, and *R_S_* values (**1** vs. **2** and **3**); (ii) the four difluoro-substituted tryptophans **4**, **5**, **6**, and **7** behave in different ways. Analyte **4** exhibited lower *k*_1_, *α*, and *R_S_* values compared to analyte **1** on the T-3.0 column, while higher *k*_1_ and lower *α* and *R_S_* values were registered on the Tag-3.0 column. Analyte **5** yielded similar *k*_1_ values but lower *α* and *R_S_* values compared to analyte **4** on both columns. For analytes **6** and **7**, a slightly lower *k*_1_ value but higher *α* and *R_S_* values were observed on both columns. It seems that the substitution with fluorine at 4,5-, 5,6- and 5,7-positions resulted in higher enantioselectivities and resolutions than did substitution in the 4,6-positions; (iii) the tetra-substituted analog (**8**) exhibited lower *k*_1_, *α*, and *R_S_* values on both columns. Either the larger size of the molecule or the modified H-binding ability of the indole N-H group (affected by the electronegative fluorine substituents) can be proposed as an explanation for the observed slight reduction in interactions in the basket of the macrocyclic glycopeptide selectors.

Results of the experiments with H_2_O/MeCN = 45/55 (*v/v*) containing 0.1 *v*% AcOH mobile phases are depicted in Table 2. It can be stated that (i) despite the similar eluent strength, in the MeCN-containing mobile phases, *k*_1_, *α*, and *R_S_* values differ considerably; in all cases, the value of *k*_1_ is higher on T-3.0, while the values of *α* and *R_S_* are much higher on the Tag-3.0 column. For all analytes, very small *R_S_* values were obtained on the T-3.0 column; (ii) in the case of analytes **1**–**3**, *k*_1_, *α*, and *R_S_* values were very similar on the T-3.0 and Tag-3.0 columns. Chromatographic data for the monofluoro-substituted analytes, similarly to the results obtained in MeOH-containing mobile phases, do not differ considerably from the data obtained for the unsubstituted Trp (**1**); (iii) for di- (**4**–**7**) and tetra-fluoro-substituted-Trp (**8**), a much smaller *k*_1_ value was observed on T-3.0 than for analytes **1**–**3**, while the values of *α* and *R_S_* do not differ considerably from those in the results obtained for analytes **1**–**3**. On Tag-3.0 CSP, *k*_1_ values were similarly low for analytes **1**–**3**, but higher *α* and *R_S_* values were registered.

**Figure 5:**
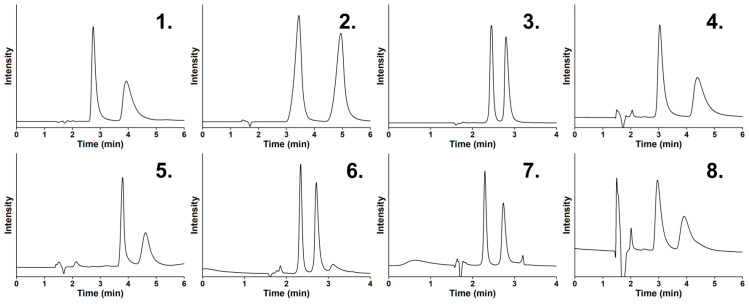
Selected chromatograms of the studied analytes. Chromatographic conditions: column, for analytes **3**, **6**, and **7**, TeicoShell; for analytes **1**, **2**, **4**, **5**, and **8**, TagShell; mobile phase, for analytes **1**, **3**, **4**, **6**, **7**, and **8**, H_2_O/MeOH = 30/70 (*v/v*) containing 0.1 *v*% AcOH‘ for analytes **2** and **5**, H_2_O/MeCN = 20/80 (*v/v*) containing 0.1 *v*% AcOH; detection, 215 nm; flow rate, 0.3 mL min^–1^; temperature, 20 °C; analytes, **1**, Trp; **2**, 5-FTrp, **3**; 6-FTrp; **4**, 4,5-diFTrp; **5**, 4,6-diFTrp; **6**, 5,6-diFTrp; **7**, 5,7-diFTrp; **8**; 4,5,6,7-tetraFTrp; enantiomeric elution order; in all cases, *L* < *D*.

In summary, fluorine substitution does not cause marked changes in the measured *k*_1_, *α*, and *R_S_* values on any of the studied columns in the MeOH-containing mobile phases. In MeCN-containing mobile phases, more significant changes could be detected on the teicoplanin-based CSP, mainly for analytes **1**–**3**. The nature of the polar modifier (MeOH vs. MeCN) has a greater influence on the chromatographic characteristics of the fluorinated tryptophans than do the presence and position of the fluorine atom(s). Utilizing MeOH-based mobile phases, all analytes could be baseline separated on both columns under the applied conditions, while in the MeCN-containing mobile phase, baseline separation could be achieved only on the Tag-3.0 column with relatively shorter retention. The enantiomeric elution order was constant under all applied conditions (*L* < *D*), so neither the eluent composition nor the fluorination led to a change in the elution order. As a representation of the enantioseparations of the studied fluorotryptophans, chromatograms are shown in Figure 5.

### 2.5. Influence of the Column Temperature and Thermodynamic Considerations

One possible way to explore the retention mechanism is to investigate the temperature dependence of the chromatographic parameters and calculate thermodynamic characteristics [38,39,40,41,42,43,44]. For this purpose, the most frequently applied approach is the van’t Hoff analysis, which predicts a linear relationship between ln *α* and *T*^−1^, as described by Equation (2) below:

(2) $$ln\;\alpha =-\frac{∆\left(∆H{}^{\circ}\right)}{RT}+\frac{∆(∆S{}^{\circ})}{R}$$

where *α* is the selectivity factor, Δ(Δ*H°*) and Δ(Δ*S°*) are the differences in standard enthalpy and standard entropy, *R* is the universal gas constant, and *T* is the temperature in degrees Kelvin. All the theoretical limitations of this simplified approach were comprehensively discussed by Asnin and Stepanova [45]. Keeping the limitations of this approach in mind, we believe that the evaluation based on chromatographic characteristics obtained under the same conditions for model compounds with significant structural analogy is a viable approach that can provide useful information to support a better understanding of the separation mechanism.

For the determination of the thermodynamic characteristics, *k*, *α*, and *R_S_* were measured for all studied analytes from 5 °C up to 50 °C on T-3.0 and TAG-3.0 CSPs, applying mobile phases of H_2_O/MeOH = 30/70 (*v/v*), and H_2_O/MeCN = 45/55 (*v/v*) containing 0.1 *v*% AcOH. The data so obtained are presented in Tables S2 and S3. The *k* values on both CSPs decreased with increasing temperature for all analytes. As usually observed, *α* decreased with increasing temperature, except for analyte **5** on the Tag-3.0 column in the H_2_O/MeOH = 30/70 (*v/v*) mobile phase, where *α* slightly increased with increasing temperature. Generally, *R_S_* also decreased with increasing temperature. However, a special behavior was registered for all analytes in the MeOH-containing mobile phase on Tag-3.0 CSP (Table S2); in all cases, *R_S_* increased with increasing temperature, probably due to the favorable kinetic effects, i.e., the adsorption-desorption processes are faster at higher temperatures, resulting in a narrower peak width and a higher *R_S_* value.

To exclude the problems related to the determination of the phase ratio [45], the differences in the changes in standard enthalpy and entropy [−Δ(Δ*H°*) and −Δ(Δ*S°*)] were calculated based on Equation (2). The data obtained in this way (presented in Table 3) can be utilized for thermodynamic characterization.

Examining the Δ(Δ*G°*) values determined for the studied CSPs [Δ(Δ*G° =* Δ(Δ*H°*) − *T×*Δ(Δ*S°*)], it can be concluded that the more negative Δ(Δ*G°*) values found for all analytes on Tag-3.0 CSP reflect more favorable bindings in both mobile-phase systems compared to the T-3.0 CSP. Analyzing the Δ(Δ*H°*) and Δ(Δ*S°*) values, it is important to highlight the difference observed between the MeOH- and MeCN-containing eluents. In the MeOH-containing mobile-phase systems, the values of Δ(Δ*S°*) are more negative on T-3.0 than on Tag-3.0 CSP, while there are no significant differences in the values of Δ(Δ*H°*). Oppositely, in the MeCN-containing mobile-phase systems, the values of both Δ(Δ*S°*) and Δ(Δ*H°*) are more negative on Tag-3.0 than on T-3.0 CSP.

It can therefore be stated that from a thermodynamic point of view, in the MeOH-containing mobile-phase systems, it is mainly entropic effects that affect selectivity, while in the MeCN-containing mobile phases, both the entropy- and enthalpy-related changes are responsible for the different binding properties of the teicoplanin and teicoplanin aglycone-based selectors. To distinguish between the enthalpic and entropic contribution to the free energy of adsorption, the enthalpy/entropy ratio, [*Q* = Δ(Δ*H°*)/[298 × Δ(Δ*S°*)], was also determined. Nearly all of the values of *Q* (shown in Table 3) were greater than 1, indicating enthalpically driven separations. The only exception was for analyte 5 on Tag-3.0 CSP in the MeOH-containing mobile phase, where entropically controlled separation was found.

Further information can be extracted from the Δ(Δ*H°*) and Δ(Δ*S°*) values, and correlations between the structure of the analyte and the thermodynamic parameters can also be explored. The fluoro-substitution of Trp in most cases does not cause a dramatic change in Δ(Δ*H°*) and Δ(Δ*S°*), except for in analytes 5 and 8, which have markedly different Δ(Δ*H°*) and Δ(Δ*S°*) values. Most probably, the fluorine substitution in the 4,6-position makes the structure of the molecule unfavorable for strong interactions in the basket of the teicoplanin and teicoplanin aglycone selector. A comparison of the effect of the mobile-phase system on thermodynamic and chromatographic parameters reveals that more negative Δ(Δ*G°*) values and baseline resolutions were obtained in the MeOH-containing mobile phases on both columns. In mobile phases containing MeCN, Δ(Δ*G°*) ranged between (−0.21)–(−0.38) kJ mol^−1^ and partial separation could be observed (*R_S_* << 0.99) on T-3.0 CSP, while Δ(Δ*G°*) ranged between (−1.08)–(−1.90) kJ mol^−1^, resulting in baseline separation on Tag-3.0 CSP. It can be concluded that the nature of the bulk solvent has a more pronounced effect on the chiral recognition than do the number and position of fluorine atoms of the tryptophan ring.

### 2.6. Influence of the Flow Rate, Kinetic Considerations

**Figure 6:**
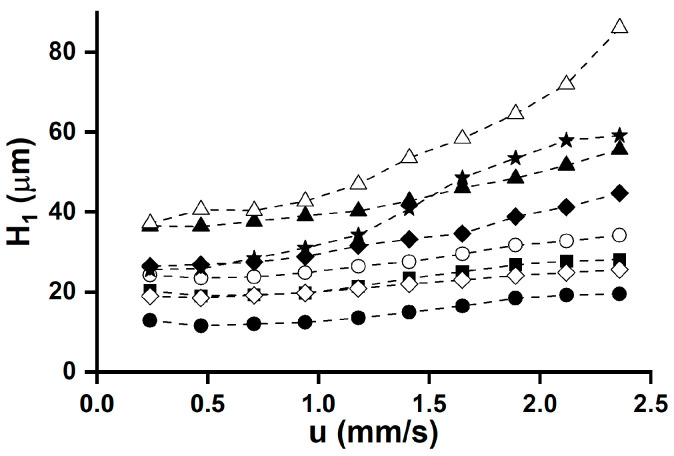
van Deemter plots of fluorinated tryptophan analogs on TeicoShell CSP. Chromatographic conditions: column, TeicoShell, T-3.0; mobile phase, H_2_O/MeCN (20/80 *v/v*) containing 0.1 *v*% AcOH; flow rate, 0.1–1.0 mL min^–1^; detection, 215 nm; temperature, 20 °C; symbols for analytes, Trp, ■; 5-FTrp, ●; 6-FTrp, 〇; 4,5-diFTrp, ▲; 4,6-diFTrp, △; 5,6-diFTrp, ◆; 5,7-diFTrp, ◇; 4,5,6,7-tetraFTrp, ★.

For the kinetic characterization of the studied CSPs, chromatographic parameters were measured, applying different flow rates of the H_2_O/MeCN = 20/80 (*v/v*) mobile phase containing 0.1 *v*% AcOH for analytes **1**, **2**, **6**, and **8** at 20 °C. Here, the mobile-phase composition was chosen to avoid high back-pressures at higher flow rates. The flow rate was varied between 0.1–1.0 mL min^–1^ (0.24–2.36 mm s^–1^) on the columns with a 3.0 mm i.d. and between 0.05–0.6 mL min^–1^ (0.24–2.89 mm s^–1^) on the columns with a 2.1 mm i.d. Enantioselectivity was not affected by the flow rate, while a significant decrease (15%–40%) was observed in the resolution over the studied flow-rate interval. For the quantitative description of the kinetics, so-called van Deemter plots were created, i.e., plate heights (*H*) were calculated based on the measured chromatographic characteristics and were plotted against the linear velocities (*u*) [17,39,40,41,42]. As illustrated in Figure 6, the plate heights measured on T-3.0 CSP are strongly dependent on the nature of the analyte, reaching a minimum at 0.5 mm/s linear velocity.

The calculated plate heights for most of the analytes were around 20 µm, which correlates well with the data published in the literature [22]. In some cases, the shape of the obtained van Deemter curves was somewhat different from the pattern usually described. Unusual kinetic characteristics have been previously reported in the literature [43,46,47]. It is worth mentioning that the narrow-bore columns showed greater kinetic efficiencies in the enantioseparation of fluorinated tryptophans, as we have earlier reported for α-substituted proline analogs [48] and certain fluorinated phenylalanine analogs [20]. However, contradictory results have also been published [20], underlining the fact that besides the physical dimensions of the columns and mobile-phase composition, the nature of the analyte has a remarkable influence on the observed kinetic characteristics.

## 3. Materials and Methods

### 3.1. Chemicals and Materials

Racemic and L-Trp were purchased from Sigma-Aldrich (St. Louis, MO, USA), while racemic and L-5-fluorotryptophan (5-FTrp) and 6-fluorotryptophan (6-FTrp) were obtained from Apollo Scientific (Whitefield Rd, Bradbury, Stockport, UK). L-4,5-difluorotryptophan (L-4,5-diFTrp), L-4,6-difluorotryptophan (L-4,6-diFTrp), L-5,6-difluorotryptophan (L-5,6-diFTrp), L-5,7-difluorotryptophan (L-5,7-diFTrp) and L-4,5,6,7-tetrafluorotryptophan (L-4,5,6,7-tetraFTrp) were synthesized by the enzyme-catalyzed reaction of fluorinated indoles (4,5-di-, 4,6-di-, 5,6-di-, 5,7-di-, 4,5,6,7-tetra-fluoroindole) and L-serine, as reported in the literature [49,50,51,52] and described in the Supporting Material.

For the synthesis of racemic 4,5-diFTrp, 4,6-diFTrp, 5,6-diFTrp, L-5,7-diFTrp, and 4,5,6,7-tetraFTrp, enzymatic racemization was carried out according to the method described in the literature [53].

Water, methanol (MeOH), acetonitrile (MeCN), formic acid (FA), and glacial acetic acid (AcOH) of LC-MS grade, ammonium formate (NH_4_HCOO), ammonium acetate (NH_4_OAc), and triethylamine (TEA) of analytical reagent grade were obtained from VWR International (Radnor, PA, USA).

### 3.2. Apparatus and Chromatography

For the liquid chromatographic measurements, a Waters^®^ ACQUITY UPLC^®^ H-Class PLUS UHPLC System was applied with Empower 3 software (Waters Incorporation, Milford, MA, USA). The UHPLC system consisted of a quaternary solvent manager, sample manager FTN-H, column manager, photodiode array detector, and QDa mass spectrometer detector.

All columns applied in this study utilize core-shell particles with a core size of 1.7 μm and a shell thickness of 0.5 μm and were obtained from AZYP, LLC (Arlington, TX, USA). The physical dimensions are 100 × 3.0 mm i.d. or 100 × 2.1 mm i.d. Abbreviations applied for the different CSPs are as follows: for internal diameter dimensions, they are 3.0 and 2.1; VancoShell columns, which use vancomycin as the selector, are referred to as V-3.0; TeicoShell columns, which use teicoplanin, are T-3.0 and T-2.1; and Tagshell columns, which use teicoplanin aglycone, are Tag-3.0, and Tag-2.1.

MeOH was used for the preparation of the stock solutions of analytes (1.0 mg mL^–1^) while further dilution was done with the eluent. The hold-up time of the columns was measured by injection of a sample consisting of 0.1% AcOH dissolved in MeOH, while the UV signal was detected at 215 nm. The flow rate was set at 0.3 mL min^–1^ (if not otherwise stated), and the column temperature was set at 20 °C (if not otherwise stated).

For the chromatographic characterization of the studied systems, the retention factor (*k*) was determined based on Equation (3),

(3) $$k=\frac{t_{r}-t_{0}}{t_{0}}$$

where *t_r_* is the retention time, while *t*_0_ is the hold-up time. Enantioselectivity was determined by Equation (4),

(4) $$\alpha =\frac{k_{2}}{k_{1}}$$

where *k*_1_ and *k*_2_ are the retention factors of the earlier- and later-eluted component, respectively. Resolution was calculated based on Equation (5),

(5) $$R_{S}=2*\frac{t_{r2}-t_{r1}}{w}$$

where *t_r_*_1_ and *t_r_*_2_ are the retention times of the earlier- and later-eluted component, respectively, while *w* is the peak width measured at baseline.

## 4. Conclusions

The liquid chromatographic enantioselective separations of fluorine-substituted tryptophan analogs of potential pharmaceutical importance were studied by the application of macrocyclic glycopeptide-based CSPs. Chromatographic characterizations were performed both in RPM and PIM; however, acceptable peak shapes were obtained only in RPM. Mobile-phase additives (organic salts or acids) had a marked influence on the peak shape and symmetry; the best performances were obtained with AcOH. When the optimized mobile-phase conditions were applied in RPM, both the teicoplanin and the teicoplanin aglycone-based selectors immobilized on core-shell particles resulted in efficient separations; all the studied analytes could be baseline-resolved, most often within a few minutes. Based on the experiments carried out with the variation of mobile-phase conditions, the chromatographic properties of the fluorotryptophans could be explored. The greater retentions observed with increasing H_2_O content can be explained by the increasing hydrophobic interactions, while the lower solubility of the fluorinated amino acids in mobile phases with high MeOH content also contributes to the observed chromatographic properties. Basically, similar chromatographic behavior was observed in the MeCN-containing mobile phases, where the aprotic MeCN resulted in a more significant enhancement in retention but, conversely, in poorer selectivities and resolutions. To describe the possible ionic interactions, the stoichiometric displacement model was utilized. Based on the relatively small slopes obtained, only a slight contribution of ionic interactions to retention can be assumed. The fluoro-substitution of the tryptophan ring had an unexpectedly small influence on the chromatographic properties; significant variations in the chromatographic properties were observed only in the MeCN-containing mobile phases. Further details of the recognition mechanism were explored by a thermodynamic characterization, where enthalpically driven enantioseparations were found for all studied analytes except for 4,6-difluorotryptophan. The chromatographic behavior of the fluorine-containing tryptophan was also recorded with varying flow rates, where higher flow rates resulted in some reduction in the resolution without significantly affecting enantioselectivity. The kinetic evaluation demonstrated the higher kinetic efficiency of the narrow-bore columns in the enantioseparation of the fluorinated tryptophan analogs.

## Figures and Tables

**Table 1:** Comparison of separation performances of TeicoShell and TagShell stationary phases in water/methanol mobile phases containing 0.1 *v*% AcOH as a mobile-phase additive.

Analyte	Column	*k*_1_	*α*	*R_S_*	*e.e.o.*
**1**	T-3.0	0.62	1.40	2.42	*L* < *D*
Trp	TAG-3.0	0.62	1.97	2.52	*L* < *D*
**2**	T-3.0	0.54	1.42	2.38	*L* < *D*
5-FTrp	TAG-3.0	0.60	2.11	2.52	*L* < *D*
**3**	T-3.0	0.60	2.10	2.20	*L* < *D*
6-FTrp	TAG-3.0	0.60	2.10	2.20	*L* < *D*
**4**	T-3.0	0.55	1.41	2.48	*L* < *D*
4,5-diFTrp	TAG-3.0	0.96	1.85	2.74	*L* < *D*
**5**	T-3.0	0.61	1.19	1.16	*L* < *D*
4,6-diFTrp	TAG-3.0	1.05	1.29	0.99	*L* < *D*
**6**	T-3.0	0.47	1.52	2.73	*L* < *D*
5,6-diFTrp	TAG	0.56	2.18	2.83	*L* < *D*
**7**	T-3.0	0.41	1.65	3.00	*L* < *D*
5,7-diFTrp	TAG-3.0	0.59	2.39	3.29	*L* < *D*
**8**	T-3.0	0.50	1.31	1.57	*L* < *D*
4,5,6,7-tetraFTrp	TAG-3.0	0.60	1.69	1.86	*L* < *D*

Chromatographic conditions: columns, TeicoShell (T-3.0) and TagShell (TAG-3.0); mobile phase, H_2_O/MeOH = 30/70 (*v/v*) containing 0.1 *v*% AcOH; flow rate, 0.3 mL min^–1^; detection, 215 nm; *e.e.o.*, enantiomeric elution order.

**Table 2:** Comparison of separation performances of TeicoShell and TagShell stationary phases in water/acetonitrile mobile phases containing 0.1 *v*% AcOH as a mobile-phase additive.

Analyte	Column	*k*_1_	*α*	*R_S_*	*e.e.o.*
**1**	T-3.0	1.41	1.08	0.23	*L* < *D*
Trp	TAG-3.0	0.32	1.65	1.37	*L* < *D*
**2**	T-3.0	1.30	1.08	0.36	*L* < *D*
5-FTrp	TAG-3.0	0.29	1.72	1.80	*L* < *D*
**3**	T-3.0	1.30	1.07	0.20	*L* < *D*
6-FTrp	TAG-3.0	0.29	1.71	1.73	*L* < *D*
**4**	T-3.0	0.56	1.21	0.75	*L* < *D*
4,5-diFTrp	TAG-3.0	0.26	1.92	2.25	*L* < *D*
**5**	T-3.0	0.55	1.09	0.31	*L* < *D*
4,6-diFTrp	TAG-3.0	0.30	1.52	1.45	*L* < *D*
**6**	T-3.0	0.50	1.08	0.25	*L* < *D*
5,6-diFTrp	TAG	0.29	1.75	1.59	*L* < *D*
**7**	T-3.0	0.43	1.16	0.99	*L* < *D*
5,7-diFTrp	TAG-3.0	0.17	2.47	2.22	*L* < *D*
**8**	T-3.0	0.43	1.11	0.57	*L* < *D*
4,5,6,7-tetraFTrp	TAG-3.0	0.23	1.89	1.66	*L* < *D*

Chromatographic conditions: columns, TeicoShell (T-3.0) and TagShell (TAG-3.0); mobile phase, H_2_O/MeCN = 45/55 (*v/v*) containing 0.1 *v*% AcOH; flow rate, 0.3 mL min^–1^; detection, 215 nm; *e.e.o.*, enantiomeric elution order.

**Table 3:** Thermodynamic parameters, Δ(Δ*H°*), Δ(Δ*S°*), *T×*Δ(Δ*S°*), Δ(Δ*G°*), correlation coefficients (*R*^2^), and *Q* values of fluorinated tryptophan analogs on TeicoShell and TagShell columns in MeOH- or MeCN-containing mobile phases.

Analyte	Eluent	−Δ(Δ*H°*) (kJ/mol)	−Δ(Δ*S°*) (J/(mol*K))	*R* ^2^	−*T×*Δ(Δ*S°*)_298K_ (kJ/mol)	−Δ(Δ*G°*)_298K_ (kJ/mol)	*Q*
H_2_O/MeOH = 30/70 (*v/v*) containing 0.1 *v*% AcOH							
**1**	T-3.0	2.86	6.61	0.998	1.97	0.89	1.45
Trp	Tag-3.0	2.74	3.13	0.990	0.93	1.81	2.94
**2**	T-3.0	3.23	6.71	0.996	2.00	1.23	1.62
5-FTrp	Tag-3.0	3.02	2.58	0.986	0.78	2.26	3.93
**3**	T-3.0	2.65	5.94	0.996	1.77	0.88	1.50
6-FTrp	Tag-3.0	2.86	3.11	0.982	0.93	1.93	3.09
**4**	T-3.0	2.66	6.01	0.978	1.79	0.87	1.49
4,5-diFTrp	Tag-3.0	2.33	2.48	0.917	0.88	1.59	3.15
**5**	T-3.0	1.38	3.10	0.975	0.92	0.46	1.50
4,6-diFTrp	Tag-3.0	−0.92	−0.59	0.983	−1.67	0.75	0.55
**6**	T-3.0	2.65	5.41	0.998	1.61	1.04	1.64
5,6-diFTrp	Tag-3.0	2.94	2.85	0.982	0.85	2.09	3.46
**7**	T-3.0	3.51	7.59	0.998	2.26	1.25	1.55
5,7-diFTrp	Tag-3.0	3.47	3.90	0.982	1.16	2.31	2.99
**8**	T-3.0	2.08	4.70	0.998	1.40	0.68	1.48
4,5,6,7-tetraFTrp	Tag-3.0	1.71	1.36	0.978	0.410	1.30	4.20
H_2_O/MeCN = 45/55 (*v/v*) containing 0.1 *v*% AcOH							
**1**	T-3.0	1.36	3.58	0.987	1.07	0.31	1.29
Trp	Tag-3.0	4.82	10.76	0.993	3.20	1.61	1.50
**2**	T-3.0	1.40	3.53	0.986	1.05	0.35	1.33
5-FTrp	Tag-3.0	5.17	10.97	0.991	3.27	1.90	1.56
**3**	T-3.0	1.50	4.03	0.983	1.20	0.30	1.26
6-FTrp	Tag-3.0	4.78	10.33	0.992	3.08	1.70	1.56
**4**	T-3.0	1.37	3.64	0.989	1.08	0.28	1.20
4,5-diFTrp	Tag-3.0	4.59	10.15	0.967	3.02	1.56	1.52
**5**	T-3.0	1.13	3.05	0.992	0.92	0.21	1.23
4,6-diFTrp	Tag-3.0	2.95	6.28	0.992	1.87	1.08	1.58
**6**	T-3.0	1.65	4.58	0.993	1.36	0.30	1.22
5,6-diFTrp	Tag-3.0	4.40	9.07	0.9900	2.70	1.69	1.63
**7**	T-3.0	1.56	4.97	0.993	1.48	0.38	1.26
5,7-diFTrp	Tag-3.0	5.45	11.90	0.992	3.55	1.90	1.54
**8**	T-3.0	1.59	4.52	0.998	1.35	0.25	1.18
4,5,6,7-tetraFTrp	Tag-3.0	1.80	5.21	0.995	1.55	1.44	1.16

Chromatographic conditions: columns, TeicoShell (T-3.0) and TagShell (Tag-3.0); mobile phase, H_2_O/MeOH = 30/70 (*v/v*) containing 0.1 *v*% AcOH and H_2_O/MeCN = 45/55 (*v/v*) containing 0.1 *v*% AcOH; flow rate, 0.3 mL min^–1^; detection, 215 nm; *Q* = Δ(Δ*H°*)/298 × Δ(Δ*S°*); temperature, 5–50 °C.

## Data Availability

All data generated or analyzed during this study are included in the article/Supplementary Materials.

## Supplementary Materials

The supporting information can be downloaded at: https://www.mdpi.com/article/10.3390/ijms25094719/s1.
