# Supplementary material for: Liquid Chromatographic Enantioseparation of Newly Synthesized Fluorinated Tryptophan Analogs Applying Macrocyclic Glycopeptides-Based Chiral Stationary Phases Utilizing Core-Shell Particles
Source: Int J Mol Sci. 2024 Apr 26;25(9):4719. doi: 10.3390/ijms25094719 (PMC11083430; doi:10.3390/ijms25094719)
Supplement: Supplementary file 1 [file ijms-25-04719-s001.zip › ijms-2962218-supplementary.pdf]

Supporting Information for

Liquid Chromatographic Enantioseparation of Newly Synthesized Fluorinated Tryptophan Analogs Applying Macrocyclic Glycopeptides-Based Chiral Stationary Phases Utilizing Core-Shell Particles

Dániel Tanács [1], Róbert Berkecz [1], Zsolt Bozsó [2], Gábor K. Tóth [2], Daniel W. Armstrong [3], Antal Péter [1] and István Ilisz [1,*]

[1] Institute of Pharmaceutical Analysis, University of Szeged, H-6720 Szeged, Hungary
[2] Department of Medical Chemistry, University of Szeged, H-6720 Szeged, Hungary
[3] Department of Chemistry and Biochemistry, University of Texas at Arlington, Arlington, TX 76019-0065, USA

Corresponding author: István Ilisz
Institute of Pharmaceutical Analysis, University of Szeged, Somogyi B. u. 4, H-6720 Szeged, Hungary
E-mail: ilisz.istvan@szte.hu

Description of the preparation of enantiopure di- and tetra-fluorinated tryptophans

Our reaction conditions were merged from those found in the literature [1-4]. 0.25 mmol L-serine and 4 mg of pyridoxal-5'-phosphate hydrate (Biosynth Carbosynth, Staad, Switzerland) were dissolved in 10 mL KH_2PO_4/K_2HPO_4 buffer (0.1 M, pH=8). 0.2 mmol fluorinated indole was dissolved in 200 µl of methanol (Lichrosolv®, Supelco®, Merck Budapest, Hungary) and added to the above-mentioned solution dropwise, while it was vigorously stirred. Finally, 2 mg of apotrypthophanase enzyme (75-150 units/mg, Sigma-Aldrich, St. Louis, MO, USA) was added to the mixture. The temperature was kept at 40 ˚C. The reaction was monitored by LC-MS and when the HPLC trace indicated it was halted (1-4 weeks, depending on the substrate) and purified by preparative RP-HPLC. The LC-MS system consisted of an Agilent (Santa Clara, CA, USA) 1200 HPLC and a Waters ACQUITY SQ detector (Milford, MA, USA). Eluent A and B were 0.1% TFA in water and 0.1% TFA in water (20%) and acetonitrile (80%) (Merck KGaA, Darmstadt, Germany), respectively. The HPLC trace was monitored at 278 and 210 nm. For analytical measurements, a Phenomenex Luna C18 column (10µm, 100Å, 250×4.6 mm i.d.) with a gradient elution and a flow rate of 1.0 ml min^{-1} was used. Purification was done on a Shimadzu (Kyoto, Japan) 20AD HPLC system on Phenomenex Luna C18 (10µm, 100Å, 250×21.2 mm i.d.) column applying a flow rate of 5.0 ml min^{-1} and detection wavelength of 210 nm. The pure fractions were pooled and lyophilized.

[1] D.R.M. Smith, T. Willemse, D.S.Gkotsi, W. Stephens, B.U.W. Maes, S. Ballet, R.J.M. Goss, Org. Lett. 16 (2014) 2622-2625.
[2] A. Shimada, H. Ozaki, T. Saito, N. Fujii, J. Chromatogr. B., 879 (2011) 3289-3295.
[3] J. Du, J.J. Duan, Q. Zhang, J. Hou, F. Bai, N. Chen, G. Bai, Appl. Biochem. Microbiol. 48 (2012) 159-166.
[4] J.M. Corr, R.M.D. Smith, R.J.M. Goss, Tetrahedron, 72 (2016) 7306-7310.

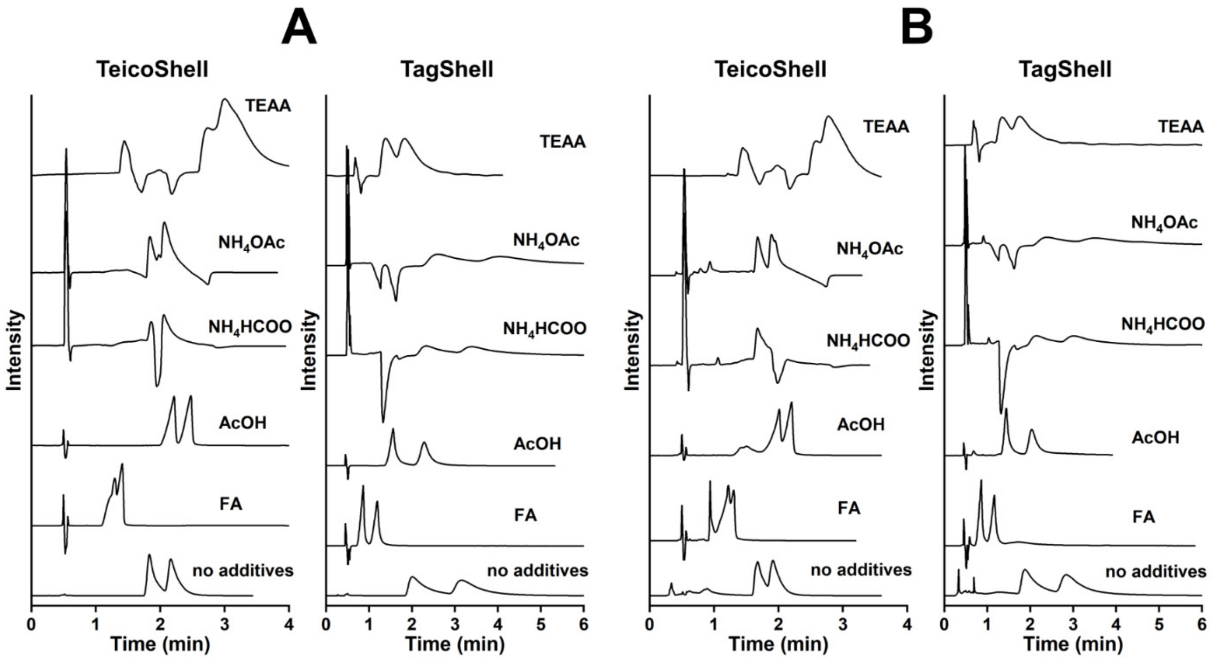

Figure S1

Effects of acid and salt additives on the chiral separations of 5-FTrp (A) and 5,6-diFTrp (B)

Chromatographic conditions: columns, TeicoShell and TagShell; mobile phase H_2O/MeOH = 85/15 (*v/v*) containing NH_4OAc, NH_4HCOO, AcOH, FA, and H_2O/MeOH = 70/30 (*v/v*) containing TEAA; the concentration of NH_4OAc and AcOH were 17.5 mM, while the concentration of NH_4HCOO and FA were 26.5 mM (all corresponding to 0.1 *v*% AcOH concentration); detection, 215 nm; flow rate, 0.3 ml min^{-1}; temperature, 20 °C

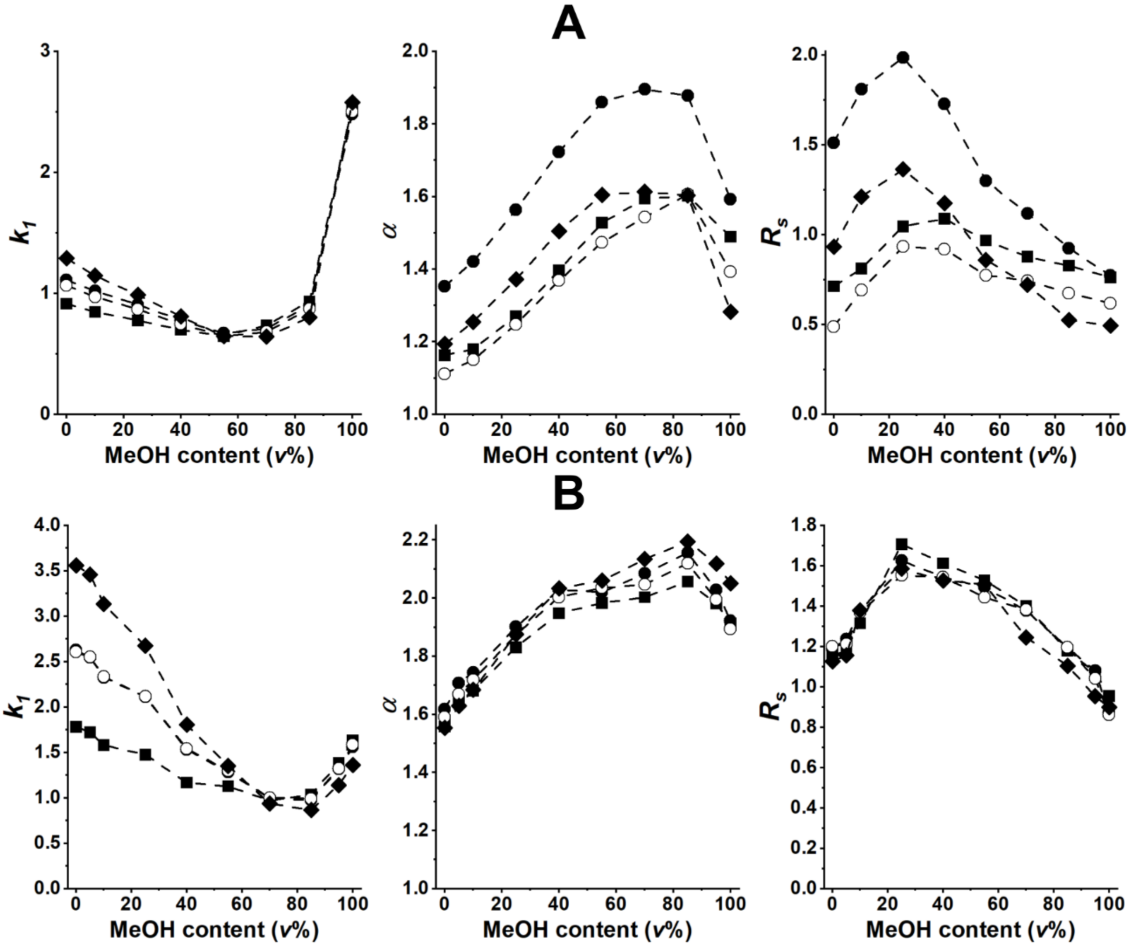

Figure S2

Effects of mobile phase composition on the chromatographic parameters applying H_2O/MeOH eluent systems with TEAA

Chromatographic conditions: **A**, TeicoShell, **B**, TagShell; mobile phase H_2O/MeOH = 100/0 – 0/100 (*v/v*) containing 0.1 *v%* TEAA; detection, 215 nm; flow rate, 0.3 ml min^{-1}; temperature, 20 °C; symbols for analytes, Trp, ■; 5-FTrp, ●; 6-FTrp, ○; 5,6-diFTrp, ◆

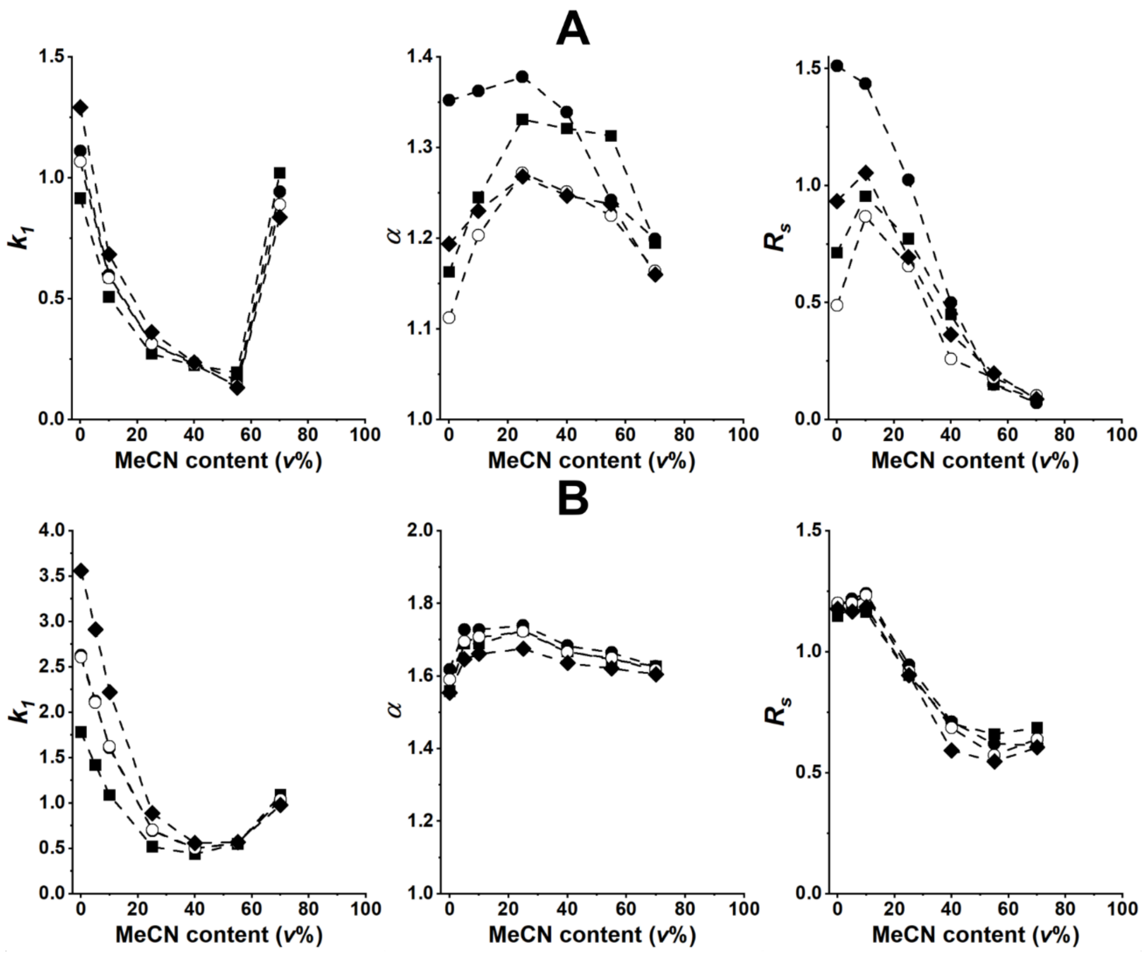

Figure S3
Effects of mobile phase composition on the chromatographic parameters applying H_2O/MeCN eluent systems with TEAA

Chromatographic conditions: **A**, TeicoShell, **B**, TagShell; mobile phase H_2O/MeCN = 100/0 – 30/70 (*v/v*) containing 0.1 *v%* TEAA; detection, 215 nm; flow rate, 0.3 ml min^{-1}; temperature, 20 °C; symbols for analytes, Trp, ■; 5-FTrp, ●; 6-FTrp, ○; 5,6-diFTrp, ◆

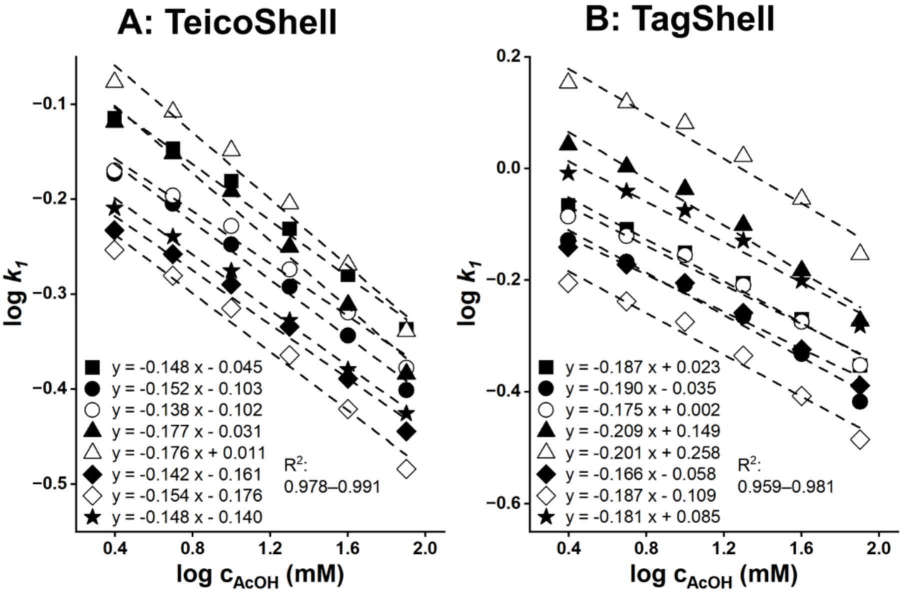

Figure S4

Effects of AcOH concentration on the retention factor of the first eluting enantiomer (*k_1*)

Chromatographic conditions: column, **A,** TeicoShell, **B**, TagShell; mobile phase H_2O/MeOH = 30/70 (*v/v*) containing 2.5, 5, 10, 20, 40 and 80 mM AcOH; detection, 215 nm; flow rate, 0.3 ml min^{-1}; temperature, 20 °C; symbols for analytes, Trp, ■; 5-FTrp, ●; 6-FTrp, ○; 4,5-diFTrp, ▲; 4,6-diFTrp, △; 5,6-diFTrp, ◆; 5,7-diFTrp, ◇; 4,5,6,7-tetraFTrp, ★

Table S1
pK values of the studied analytes

Sample	*pK* of the carboxyl group	*pK* of the amino group
1. Trp	2.51	9.54
2. 5-FTrp	2.70	9.55
3. 6-FTrp	2.70	9.54
4. 4,5-diFTrp	3.10	9.52
5. 4,6-diFTrp	3.11	9.51
6. 5,6-diFTrp	3.05	9.56
7. 5,7-diFTrp	3.13	9.56
8. 4,5,6,7-tetraFTrp	4.21	9.54

The *pK* values of the studied analytes were calculated with Marvin Sketch v. 17.29 software, ChemAxon, Budapest

Table S2

Temperature dependence of the retention factor of the first eluting enantiomer (k_1), separation factor (α), and resolution (R_S) on TeicoShell (**T-3.0**) and TagShell (**Tag-3.0**) CSPs for analytes **1-8** in H_2O/MeOH = 30/70 (*v/v*) mobile phase in the presence of AcOH as a mobile phase additive

Analyte	k_1 / α / R_S	Temperature (C°)					
		**5**	**10**	**20**	**30**	**40**	**50**
		**T-3.0**					
**1**	k_1	0.61	0.59	0.54	0.49	0.45	0.41
	α	1.56	1.53	1.45	1.41	1.36	1.31
	R_S	3.26	2.71	2.66	2.60	2.34	2.05
**2**	k_1	0.52	0.50	0.46	0.43	0.39	0.36
	α	1.79	1.77	1.68	1.61	1.55	1.48
	R_S	3.87	3.77	3.42	3.46	3.16	2.76
**3**	k_1	0.54	0.52	0.47	0.43	0.39	0.36
	α	1.55	1.50	1.44	1.40	1.35	1.32
	R_S	2.95	2.63	2.63	2.49	2.19	1.87
**4**	k_1	0.60	0.57	0.52	0.47	0.43	0.39
	α	1.56	1.50	1.43	1.39	1.35	1.31
	R_S	2.82	2.36	2.41	2.42	2.16	1.86
**5**	k_1	0.67	0.64	0.57	0.51	0.46	0.41
	α	1.26	1.23	1.21	1.19	1.18	1.15
	R_S	1.27	1.24	1.22	1.12	0.87	0.56
**6**	k_1	0.46	0.44	0.41	0.37	0.34	0.31
	α	1.64	1.60	1.55	1.50	1.45	1.40
	R_S	2.85	2.88	2.85	2.55	2.30	1.97
**7**	k_1	0.43	0.41	0.38	0.35	0.32	0.29
	α	1.83	1.78	1.70	1.62	1.55	1.47
	R_S	3.12	3.08	3.17	2.91	2.56	2.19
**8**	k_1	0.49	0.47	0.42	0.37	0.33	0.30
	α	1.39	1.37	1.34	1.30	1.26	1.23
	R_S	1.82	1.81	1.66	1.50	1.27	1.03
		**Tag-3.0**					
**1**	k_1	0.77	0.71	0.63	0.55	0.48	0.42
	α	2.24	2.19	2.12	2.05	1.97	1.59
	R_S	2.40	2.62	2.81	2.96	3.27	3.34
**2**	k_1	0.66	0.62	0.55	0.49	0.42	0.37
	α	2.70	2.63	2.55	2.48	2.35	2.23
	R_S	2.93	3.09	3.35	3.66	3.89	3.97
**3**	k_1	0.76	0.70	0.62	0.54	0.47	0.41
	α	2.36	2.31	2.23	2.17	2.07	1.97
	R_S	2.45	2.67	2.85	3.09	3.29	3.36
**4**	k_1	1.02	0.93	0.80	0.70	0.59	0.50
	α	2.00	1.99	1.99	1.98	1.93	1.90
	R_S	2.24	2.28	2.74	3.19	3.41	3.72

Table S2 (continued)
Temperature dependence of the retention factor of the first eluting enantiomer (k_1), separation factor (α), and resolution (R_S) on TeicoShell (**T-3.0**) and TagShell (**Tag-3.0**) CSPs for analytes **1-8** in H_2O/MeOH = 30/70 (*v/v*) mobile phase in the presence of AcOH as a mobile phase additive

Analyte	k_1<br>α<br>R_S	Temperature (C°)					
		5	10	20	30	40	50
**5**	k_1	1.38	1.24	1.04	0.87	0.72	0.59
	α	1.32	1.33	1.35	1.37	1.38	1.39
	R_S	0.67	0.88	1.01	1.20	1.62	1.79
**6**	k_1	0.67	0.63	0.55	0.49	0.42	0.37
	α	2.52	2.46	2.38	2.31	2.21	2.09
	R_S	2.57	2.63	2.88	3.12	3.48	3.46
**7**	k_1	0.56	0.53	0.47	0.41	0.36	0.32
	α	2.79	2.72	2.61	2.53	2.39	2.25
	R_S	2.64	2.80	3.10	3.10	3.48	3.41
**8**	k_1	0.97	0.90	0.75	0.65	0.55	0.47
	α	1.79	1.75	1.75	1.71	1.65	1.60
	R_S	1.52	1.56	1.87	1.99	2.20	2.18

Chromatographic conditions: columns, TeicoShell (**T-3.0**) and TagShell (**TAG-3.0**); mobile phase, H_2O/MeOH = 30/70 (*v/v*) containing 0.1 *v%* AcOH; detection, 215 nm; flow rate, 0.3 ml min^{-1}; temperature range, 5–50 °C

Table S3

Temperature dependence of the retention factor of the first eluting enantiomer (k_1), separation factor (α), and resolution (R_S) on TeicoShell (**T-3.0**) and TagShell (**Tag-3.0**) CSPs for analytes **1-8** in H_2O/MeCN = 45/55 (*v/v*) mobile phase in the presence of AcOH as a mobile phase additive

**Analyte**	k_1 α R_S	**Temperature (C°)**					
		**5**	**10**	**20**	**30**	**40**	**50**
		**T-3.0**					
**1**	k_1	0.57	0.55	0.53	0.51	0.49	0.47
	α	1.19	1.16	1.14	1.12	1.11	1.09
	R_S	1.55	1.17	0.89	0.62	0.48	0.35
**2**	k_1	0.50	0.49	0.47	0.45	0.43	0.41
	α	1.21	1.18	1.16	1.14	1.12	1.11
	R_S	1.55	1.29	1.02	0.90	0.78	0.66
**3**	k_1	0.49	0.48	0.46	0.44	0.42	0.41
	α	1.18	1.16	1.13	1.12	1.10	1.07
	R_S	1.37	1.09	0.80	0.64	0.51	0.39
**4**	k_1	0.55	0.55	0.52	0.50	0.49	0.45
	α	1.17	1.15	1.13	1.11	1.09	1.07
	R_S	1.45	1.12	0.85	0.67	0.52	0.40
**5**	k_1	0.54	0.53	0.51	0.49	0.46	0.44
	α	1.12	1.12	1.09	1.08	1.06	1.05
	R_S	1.07	0.80	0.54	0.40	0.29	0.15
**6**	k_1	0.45	0.44	0.42	0.40	0.38	0.37
	α	1.19	1.17	1.14	1.12	1.10	1.07
	R_S	1.11	0.87	0.59	0.50	0.35	0.22
**7**	k_1	0.43	0.43	0.41	0.39	0.37	0.36
	α	1.23	1.21	1.18	1.15	1.12	1.10
	R_S	1.45	1.16	0.92	0.78	0.66	0.57
**8**	k_1	0.43	0.43	0.41	0.39	0.37	0.36
	α	1.16	1.14	1.12	1.09	1.06	1.04
	R_S	1.12	0.84	0.64	0.52	0.40	0.31
		**Tag-3.0**					
**1**	k_1	0.27	0.27	0.26	0.26	0.26	0.25
	α	2.22	2.09	1.98	1.87	1.75	1.64
	R_S	1.76	1.80	1.78	1.76	1.60	1.40
**2**	k_1	0.24	0.24	0.23	0.22	0.22	0.22
	α	2.52	2.36	2.24	2.11	1.95	1.82
	R_S	2.00	1.98	1.98	1.92	1.76	1.55
**3**	k_1	0.27	0.26	0.25	0.24	0.24	0.24
	α	2.29	2.17	2.06	1.95	1.82	1.70
	R_S	1.88	1.83	1.84	1.77	1.63	1.44
**4**	k_1	0.31	0.31	0.30	0.29	0.28	0.28
	α	2.13	2.04	1.96	1.86	1.75	1.58
	R_S	1.73	1.82	1.83	1.80	1.66	1.48

Table S3 (continued)

Temperature dependence of the retention factor of the first eluting enantiomer (k_1), separation factor (α), and resolution (R_S) on TeicoShell (**T-3.0**) and TagShell (**Tag-3.0**) CSPs for analytes **1-8** in H_2O/MeCN = 45/55 (*v/v*) mobile phase in the presence of AcOH as a mobile phase additive

**Analyte**	***k₁* / *α* / *R*ₛ**	**Temperature (C°)**					
		**5**	**10**	**20**	**30**	**40**	**50**
**5**	k_1	0.36	0.36	0.34	0.32	0.30	0.29
	α	1.69	1.64	1.60	1.53	1.45	1.41
	R_S	1.18	1.19	1.20	1.18	1.06	0.93
**6**	k_1	0.26	0.26	0.24	0.23	0.23	0.23
	α	2.24	2.15	2.06	1.95	1.82	1.71
	R_S	1.73	1.76	1.80	1.77	1.60	1.41
**7**	k_1	0.22	0.22	0.21	0.21	0.21	0.20
	α	2.51	2.40	2.26	2.11	1.93	1.80
	R_S	1.80	1.77	1.81	1.76	1.50	1.34
**8**	k_1	0.29	0.29	0.28	0.26	0.26	0.25
	α	2.02	1.95	1.85	1.76	1.65	1.56
	R_S	1.45	1.38	1.37	1.34	1.27	1.11

Chromatographic conditions: column, TeicoShell (**T-3.0**) and TagShell (**TAG-3.0**); mobile phase, H_2O/MeCN = 45/55 (*v/v*) containing 0.1 *v%* AcOH; detection, 215 nm; flow rate, 0.3 ml min^{-1}; temperature range, 5–50 °C
